# Valor Preditivo do Índice de Inflamação Imune Sistêmica para a Recorrência de Pericardite Aguda Idiopática

**DOI:** 10.36660/abc.20250437

**Published:** 2026-03-18

**Authors:** Tuba Unkun, Busra Guvendi Sengor

**Affiliations:** 1 Department of cardiology Kartal Kosuyolu Education and Research Hospital Istanbul Turquia Department of cardiology, Kartal Kosuyolu Education and Research Hospital, Istanbul – Turquia

**Keywords:** Índice de Inflamação Imune Sistêmica, Pericardite, Proteína C-Reativa

## Abstract

**Fundamentos:**

Identificar pacientes com risco aumentado de recorrência de pericardite aguda continua sendo um desafio. A Proteína C-Reativa Ultrassensível (PCR-us) pode ser utilizada para prever recorrência, mas apresenta algumas limitações.

**Objetivos:**

Este estudo teve como objetivo avaliar a associação entre um novo biomarcador, o Índice de Inflamação Imune Sistêmica (SII), e a recorrência da pericardite aguda.

**Métodos:**

Este estudo de coorte retrospectivo incluiu 110 pacientes diagnosticados com pericardite aguda idiopática. O desfecho primário foi investigar a recorrência da pericardite durante o período de acompanhamento de 18 meses. Os pacientes foram divididos em dois grupos, de acordo com o desenvolvimento ou não de recorrência. Foram avaliados exame físico, eletrocardiograma, ecocardiograma e resultados laboratoriais desses pacientes. Valores de p < 0,05 foram considerados estatisticamente significativos.

**Resultados:**

A idade média dos pacientes foi de 42,8 ± 15,2 anos, e 42,7% eram do sexo masculino. O grupo com recorrência de pericardite (RP) apresentou contagens de leucócitos significativamente mais altas (8,7 ± 3,2 vs. 12 ± 4,7; p = 0,001), contagens de neutrófilos (5,4 ± 2,7 vs. 9 ± 4,5; p < 0,001), níveis de PCR-us (29,1 ± 40,9 vs. 49,5 ± 62,2; p = 0,048) e relação neutrófilo/linfócito (2,9 ± 2,5 vs. 6 ± 2,8; p < 0,001). O SII (783,2 ± 770,2 vs. 1664,2 ± 1086,6; p < 0,001) também foi significativamente mais elevado nos pacientes com recorrência. Tanto o SII quanto a PCR-us mostraram associação independente com a recorrência (p < 0,001 e p < 0,05, respectivamente).

**Conclusão:**

O SII e a PCR-us foram identificados como preditores independentes de recorrência em pacientes com pericardite aguda durante o acompanhamento de 18 meses. Assim como a PCR-us, o SII pode ser utilizado como um parâmetro importante na predição da pericardite recorrente.

## Graphical abstract

**Figura Central:**
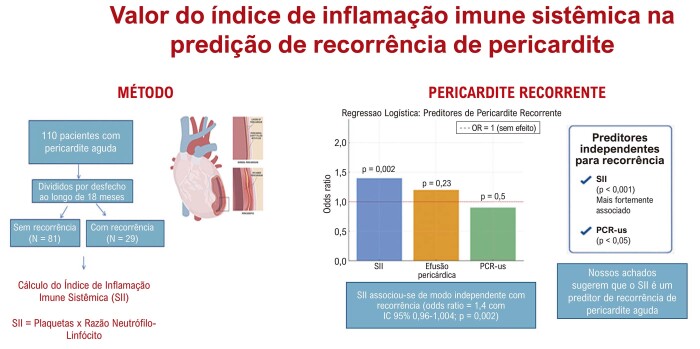
Valor Preditivo do Índice de Inflamação Imune Sistêmica para a Recorrência de Pericardite Aguda Idiopática Arq Bras Cardiol. 2026; 123(2):e20250437

## Introdução

Pericardite aguda é a doença pericárdica mais comum observada na prática clínica.^1 , 2^ Para o diagnóstico, são necessários pelo menos dois dos seguintes quatro critérios: dor torácica, atrito pericárdico, achados eletrocardiográficas anormais – como depressão do segmento PR e elevação difusa do segmento ST – e derrame pericárdico novo ou em progressão. Achados adicionais de suporte incluem marcadores inflamatórios elevados, como Proteína C-Reativa Ultrassensível (PCRus) e velocidade de hemossedimentação (VHS), bem como a detecção de inflamação por técnicas de imagem, como ressonância magnética cardíaca (RMC) ou tomografia computadorizada.^3^ As complicações da pericardite incluem tamponamento cardíaco, pericardite constritiva e, mais comumente, pericardite recorrente.^4 , 5^ A pericardite aguda é uma condição inflamatória que afeta o pericárdio. Níveis elevados de PCR-us têm sido associados à recorrência da pericardite e a desfechos cardíacos adversos.^6 , 7^ A busca por biomarcadores confiáveis, capazes de prever a recorrência na prática clínica, ainda está em andamento.

Recentemente, o Índice de Inflamação Imune Sistêmica (SII, do inglês *Systemic Immune-Inflammation Index* ), um indicador simples e abrangente, tem ganhado destaque.^8^ O SII reflete o estado inflamatório e imunológico geral ao incorporar três biomarcadores: plaquetas, neutrófilos e linfócitos.^9^ O índice é comumente utilizado para prever a presença e o prognóstico de diversas neoplasias.^10^ Estudos recentes também demonstraram que o SII está associado ao prognóstico clínico em uma variedade de doenças cardiovasculares.^1 1^ No entanto, a relação entre o SII e o prognóstico de pacientes com pericardite aguda ainda não foi estabelecida. O objetivo deste estudo é investigar a associação entre a recorrência da pericardite após um episódio agudo e o índice SII.

## Método

### População e desenho do estudo

**Figura 1:**
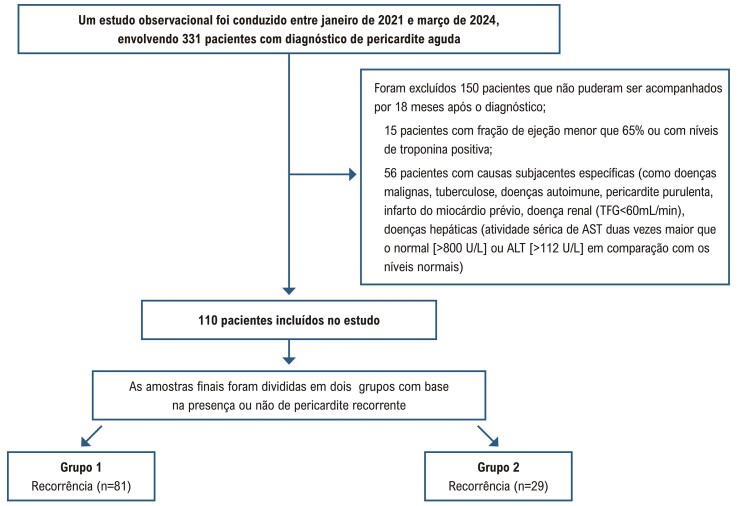
– Diagrama de fluxo da população estudada com pericardite aguda.

Este estudo do tipo coorte retrospectivo foi conduzido no *Kartal Koşuyolu High Specialty Training and Research Hospital* , Istambul, Turquia, entre janeiro de 2021 e março de 2024. O estudo envolveu 331 pacientes diagnosticados com pericardite aguda. Cento e cinquenta pacientes foram excluídos devido ao tempo insuficiente de acompanhamento após o diagnóstico. Quinze pacientes com fração de ejeção (FE) inferior a 53–54% (<53% para homens e <54% para mulheres)^3^ ou níveis positivos de troponina, e 56 pacientes com causas específicas subjacentes (como neoplasias, tuberculose, doenças autoimunes, pericardite purulenta, lesão pós-miocárdica ou pós-infarto do miocárdio, insuficiência renal [Taxa de Filtração Glomerular, TFG <60 mL/min], distúrbios hepáticos [atividade sérica de Aspartato Aminotransferase (AST) duas vezes acima do limite normal e >80 U/L ou Alanina Aminotransferase (ALT) >112 U/L. Após as exclusões, o grupo de estudo consistiu em 110 pacientes diagnosticados com pericardite aguda ( Figura 1 ). O estudo foi autorizado pelo comitê de ética local.

Todos os dados clínicos e demográficos, incluindo idade, sexo e histórico médico, foram obtidos do sistema digital de prontuários do hospital. Todos os pacientes apresentaram seu primeiro episódio de pericardite, e o diagnóstico foi estabelecido com base na presença de pelo menos dois dos quatro critérios descritos nas diretrizes mais recentes: dor torácica pericárdica, anormalidades eletrocardiográficas (nova elevação difusa do segmento ST ou depressão do PR), derrame pericárdico e atrito pericárdico.^3^ Os eletrocardiogramas de admissão, radiografias de tórax, ecocardiogramas e resultados de exames laboratoriais foram coletados dos registros hospitalares. Os exames laboratoriais incluíram hemograma completo, PCR-us, troponina I de alta sensibilidade, bioquímica sérica, testes de função tireoidiana e rastreamento para doenças específicas (como doenças autoimunes e tuberculose). A Razão Neutrófilo-Linfócito (RNL) foi calculada como a razão entre a contagem de neutrófilos e linfócitos em amostras de sangue periférico. O SII foi calculado como SII = PLT × RNL.^9^ As contagens sanguíneas completas foram determinadas utilizando um autoanalisador (Horiba ABX Diagnostics, França), e os níveis de Proteína C-Reativa foram medidos pelo método nefelométrico (Beckman Coulter).

As imagens ecocardiográficas foram obtidas do sistema digital de prontuários do hospital. O diagnóstico de tamponamento cardíaco foi estabelecido com base em uma combinação de sintomas clínicos (como pulso paradoxal, hipotensão e taquicardia) juntamente com evidências ecocardiográficas. A pericardiocentese foi realizada em pacientes com derrame pericárdico grave acompanhado de instabilidade hemodinâmica ou quando os achados clínicos sugeriam pericardite bacteriana ou neoplásica.

De acordo com as recomendações das diretrizes, Anti-Inflamatórios Não Esteroides (AINEs) ou aspirina foram utilizados como opção de tratamento de primeira linha na dose máxima tolerada.^3 , 1 2^ A dose máxima foi administrada por 1-2 semanas e, caso os níveis de PCR-us se normalizassem, a dose era reduzida gradualmente ao longo de 3-4 semanas. Em todos os casos, colchicina 0,5 mg duas vezes ao dia foi prescrita por 3 meses, com ajustes baseados na depuração de creatinina, peso corporal e idade.^3 , 1 3^ Nenhum paciente recebeu terapia com corticosteroides. A pericardite recorrente foi definida como a presença de dor torácica recorrente associada a pelo menos um dos seguintes sinais: febre, atrito pericárdico, anormalidades eletrocardiográficas, evidência de derrame pericárdico, aumento da contagem de leucócitos ou elevação de PCR-us.^3^

Todos os pacientes incluídos no estudo foram acompanhados por aproximadamente 18 meses a partir do diagnóstico. Cerca de 10 dias após a alta, foram acompanhados mensalmente durante os 3 meses seguintes e, posteriormente, a cada 3 meses até completar 18 meses. As consultas clínicas incluíram exame físico, exames laboratoriais (hemograma completo e PCR), ECG e avaliações ecocardiográficas. O desfecho do estudo foi a ocorrência da primeira recorrência de pericardite. Os pacientes foram divididos em dois grupos com base no desenvolvimento ou não de pericardite recorrente.

### Análise estatística

O estudo foi aprovado pelo comitê de ética local e esteve em conformidade com os princípios da Declaração de Helsinque. As análises estatísticas foram realizadas utilizando o *Statistical Package for the Social Sciences* (SPSS; Versão 26.0, IBM). As variáveis contínuas foram apresentadas como média e desvio padrão, devido à normalidade dos dados. As variáveis categóricas foram apresentadas em frequências absolutas e relativas. A suposição de normalidade dos dados foi avaliada pelo teste de Kolmogorov–Smirnov. A comparação entre os grupos foi realizada utilizando o teste *t* de Student para amostras independentes ou o teste do qui-quadrado, conforme apropriado. O modelo *Enter* foi utilizado na regressão logística múltipla. Os resultados desses modelos foram interpretados em termos de razões de chances ajustadas ( *aORs* ) com intervalos de confiança de 95% (IC95%). A análise da curva ROC ( *Receiver Operating Characteristic* ) foi utilizada para determinar o ponto de corte ideal de PCR-us e SII na recorrência da pericardite. Os resultados foram avaliados com intervalo de confiança de 95%, e valores de p < 0,05 foram considerados estatisticamente significativos.

## Resultados

Os pacientes foram divididos em dois grupos de acordo com a recorrência. A população do estudo consistiu em 110 pacientes (47 homens, 42,7%), com idade média de 42,8 ± 15,2 anos. As características dos pacientes estão apresentadas na Tabela 1 .

**Tabela 1:** – Características demográficas e clínicas dos pacientes

Variáveis		Sem recorrência (n=81)	Com recorrência (n=29)	Valor p
Idade (anos)		42,19 ±15,5	42 ±15,2	0,956^1^
Gênero (n)	Masculino	34	13	0,790^2^
	Feminino	47	16	
ECG (n)	Sem alteração	21	9	0,596^2^
	Com alteração	60	20	
Efusão pericárdica (n)	Leve	66 (81,4%)	15 (51,7%)	0,006*
	Moderada	4 (4,9%)	5 (17,2%)	
	Grave	11 (13,7%)	9 (31,1%)	
Presença de dor torácica (n)		70	27	0,105^2^
Atrito pericárdico (n)		17	2	0,065^2^

^1^Teste t para amostras independentes; ^2^Teste do qui-quadrado, *p<0,05; ECG: eletrocardiograma.

Todos os pacientes receberam o mesmo tratamento (AINEs e colchicina). Não houve diferença entre os grupos em relação à idade, sexo, dor torácica e alterações eletrocardiográficas. As contagens de leucócitos e neutrófilos foram mais elevadas no grupo com recorrência (p<0,001; Tabela 2 ). SII, RNL e PCR-us também foram significativamente mais altos nos pacientes com recorrência (p<0,001; p<0,001; p=0,048). Outros achados estão apresentados na Tabela 2 .

**Tabela 2:** – Comparação das variáveis laboratoriais entre os pacientes com e sem recorrência

Variáveis	Sem recorrência (n=81)	Com recorrência (n=29)	Valor p
PCR-us (mg/L)	29,1 ±40,9	49,5 ±62,2	0,048
Leucócitos (células/µL)	8,7 ±3,2	12 ±4,7	0,001*
Hemoglobina (g/dL)	13,2 ±1,7	12,8±2	0,306
Plaquetas (células/µL)	267,4 ±86,8	312,2 ±126,3	0,086
Neutrófilos (células/µL)	5,4± 2,7	9 ± 4,5	<0,001**
Linfócitos (células/µL)	2,3±1,13	2,5±3,2	0,786
SII	783,2 ± 770,2	1664,2 ±1086,6	<0,001**
Relação Neutrófilo/Linfócito	2,9 ± 2,5	6 ± 2,8	<0,001**

PCR: Proteína C-Reativa; Teste t para amostras independentes, *p<0.05, **p<0.001. SII: Índice de Inflamação Imunológica Sistêmica.

Na análise de regressão, as variáveis de confusão incluíram SII, derrame pericárdico e PCR-us. O SII esteve associado de forma independente à recorrência [Razão de Chances (OR) = 1,4; IC95% (0,96-1,54); p=0,002]. Outras variáveis estão apresentadas na Tabela 3 .

**Tabela 3:** – Análise de regressão logística para prever recorrência

Variáveis	Odds Ratio	IC95%	Valor p
SII	1,4	0,96-1,54	0,002
Efusão Pericárdica	1,2	0,78-2,7	0,23
PCR-us	0,9	0,98-1,009	0,8

*SII: Índice de Inflamação Imunológica Sistêmica; PCR-us: Proteína C-Reativa Ulltrassensível*

**Figura 2:**
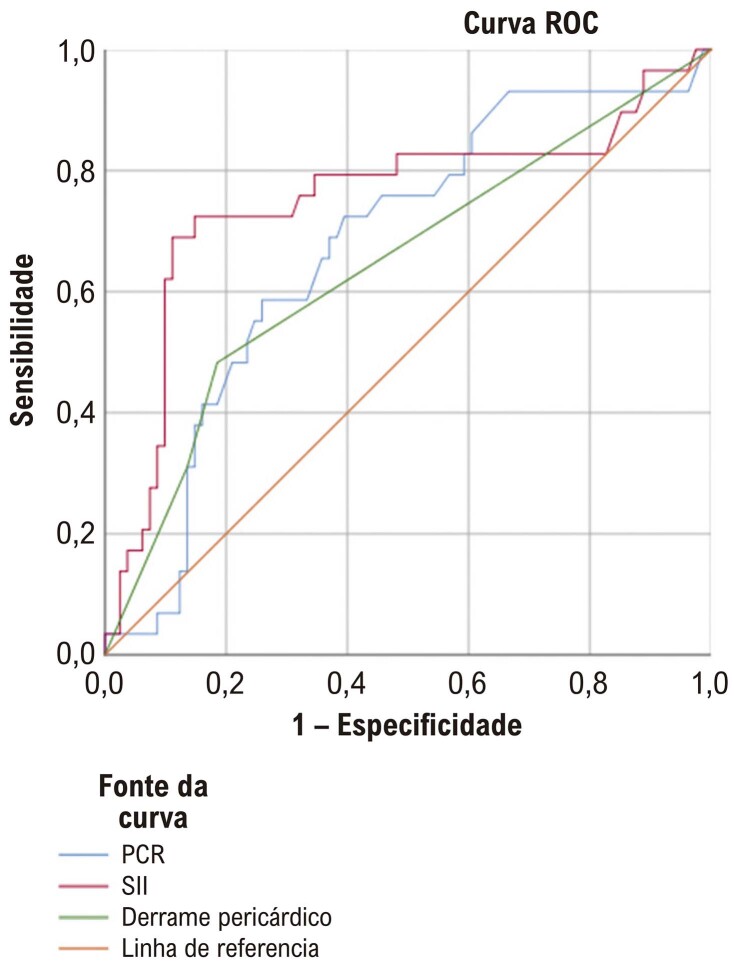
– Análise da curva ROC (Receiver Operating Characteristic).

Na análise ROC ( Figura 2 ), o SII foi identificado como o parâmetro mais associado à recorrência. O ponto de corte determinado para o SII no diagnóstico de recorrência foi 462,5, com sensibilidade de 82,8% e especificidade de 40%. Os resultados da análise ROC estão mostrados na Figura 2 (AUC: 0,751; IC: 0,630–0,872; p=0,001).

## Dıscussão

Nosso estudo demonstrou que o SII é um parâmetro simples e de baixo custo para prever a recorrência em pacientes com pericardite aguda. A pericardite aguda é a manifestação mais frequente das doenças pericárdicas e está associada a significativa morbidade. A pericardite recorrente, a complicação mais comum após a pericardite aguda, tem impacto substancial na qualidade de vida dos pacientes.^3 , 14^ Identificar indivíduos com risco de recorrência continua sendo um grande desafio clínico.

Na prática clínica, identificar pacientes com alto risco de recorrência após um primeiro episódio de pericardite aguda é fundamental, já que a pericardite recorrente leva a ataques repetidos e à progressão para doença crônica.^15^ No entanto, atualmente não há marcador específico ou processo diagnóstico para detectar esses pacientes. Indivíduos com risco aumentado de recorrência podem se beneficiar de acompanhamento mais próximo e tratamento médico mais intensivo. Além disso, modalidades avançadas de imagem, como a RMC, podem ser úteis antes da suspensão da terapia. Com sua capacidade de demonstrar inflamação tecidual persistente, a RMC pode auxiliar na determinação da duração do tratamento.^3^

Pacientes com alto risco de recorrência após um primeiro episódio de pericardite aguda não foram adequadamente investigados até o momento. De acordo com estudos anteriores, o uso de colchicina durante o ataque inicial demonstrou ser o fator mais eficaz na redução da recorrência. Por outro lado, o uso de corticosteroides, etiologias específicas da pericardite aguda, tratamento inadequado e a presença de derrame pericárdico foram todos associados a maiores taxas de recorrência.^3 , 5 , 16 - 20^

Marcadores inflamatórios são utilizados para prever a recorrência na pericardite aguda. A PCR-us pode ser usada tanto para o diagnóstico quanto para avaliar a resposta ao tratamento.^6 , 21^ Imazio et al.^7^ demonstraram que a elevação persistente da PCR-us pode prever a recorrência da pericardite. Estudos indicaram que o NLR, outro marcador inflamatório, é mais eficaz que a PCR-us na predição da recorrência da pericardite.^2 2^ Um estudo recente mostrou que o D-dímero é um biomarcador potencial para avaliação prognóstica na pericardite aguda e complicada.^2 3^ Em outro estudo, o Infla Score — desenvolvido pela combinação de diversos marcadores inflamatórios — mostrou-se eficaz na predição da recorrência da pericardite.^24^ O SII tem se mostrado, em estudos, um marcador inflamatório mais potente do que NLR ou plaqueta isoladamente, com melhor valor prognóstico.^25^ Neste estudo, verificamos que o SII esteve mais significativamente associado à probabilidade de recorrência da pericardite do que NLR, plaqueta e PCR-us isoladamente, sugerindo que o SII pode servir como um preditor mais confiável do estado inflamatório dos pacientes, resultando em maior confiabilidade na predição da recorrência da pericardite.

Hipotetizamos que o uso do SII – um índice simples, mais abrangente e menos variável, que incorpora simultaneamente múltiplos biomarcadores inflamatórios – pode servir como parâmetro complementar à PCR, rotineiramente utilizado no acompanhamento, e também fornecer valor prognóstico significativo na detecção de inflamação ativa subjacente na pericardite aguda e recorrente. Composto por neutrófilos, linfócitos e plaquetas, o SII foi recentemente desenvolvido como biomarcador abrangente de inflamação sistêmica.^9^ Originalmente, o SII foi identificado como um fator prognóstico poderoso em pacientes com carcinoma hepatocelular.^8^ Atualmente, o SII é amplamente empregado como marcador preditivo e prognóstico em diversas doenças, incluindo câncer, doenças cardiovasculares e renais.^26 - 28^

O SII ganhou popularidade nos últimos anos devido ao seu papel como marcador inflamatório, semelhante à PCR-us. Trata-se de um parâmetro simples, adequado para uso rotineiro, e sua variabilidade é menor do que a da PCR-us. Em nosso estudo, o SII foi identificado como um preditor independente de pericardite recorrente, sugerindo que pode servir como um parâmetro valioso na prática clínica. Quando utilizado em conjunto com a PCR-us, em vez de como substituto, o SII pode melhorar a predição de pacientes em risco de recorrência da pericardite.

Em nosso conheciento, não foram conduzidas pesquisas em indivíduos com pericardite aguda para explorar a associação entre o SII e o prognóstico. Em nosso estudo, o SII superou a PCR-us na predição da recorrência da pericardite.

Este estudo apresenta várias limitações. Em primeiro lugar, o tamanho relativamente pequeno da amostra, composta por 110 pacientes, pode limitar a generalização dos achados para cenários clínicos mais amplos e populações mais diversas. Além disso, o desenho retrospectivo do estudo implicou que certas variáveis – como adesão ao tratamento, duração dos sintomas e consistência do acompanhamento – não pudessem ser totalmente padronizadas ou controladas. A ausência de medições seriadas dos parâmetros laboratoriais também representa uma limitação significativa.

Adicionalmente, apenas pacientes com pericardite isolada foram incluídos. A miopericardite e a perimiocardite, que envolvem inflamação concomitante do pericárdio e miocárdio, podem apresentar sintomas clínicos e achados iniciais semelhantes, aumentando o risco de classificação incorreta na ausência de exames de imagem avançados. Contudo, como técnicas avançadas de imagem (por exemplo, a RMC) não estão rotineiramente disponíveis em todos os cenários clínicos, esses pacientes foram excluídos. Embora essa abordagem tenha permitido a seleção de uma população de estudo mais homogênea e de menor risco, ela limita a generalização dos resultados para todo o espectro das doenças pericárdicas. Além disso, de um total de 331 pacientes, apenas 110 que atenderam aos critérios de inclusão predefinidos e que possuíam dados clínicos e de acompanhamento suficientes foram incluídos na análise. Como dados clínicos, laboratoriais ou de tratamento detalhados não foram coletados de forma sistemática para os 221 pacientes excluídos, não foi possível realizar uma análise separada desse grupo. Isso pode ter introduzido um viés de seleção e reduzido a validade externa do estudo.

Por fim, embora todos os pacientes tenham recebido prescrição de AINEs e colchicina na alta, em conformidade com as diretrizes da *European Society of Cardiology* , a adesão ao tratamento após a alta não pôde ser monitorada de forma sistemática devido ao caráter retrospectivo do estudo. Métodos comumente empregados em ensaios clínicos randomizados, como acompanhamento telefônico, entrevistas presenciais ou contagem de comprimidos, não foram aplicados. Consequentemente, a uniformidade do tratamento e a avaliação da eficácia terapêutica representam limitações adicionais.

O SII foi identificado como um preditor independente para recorrência de pericardite em pacientes com pericardite aguda durante o acompanhamento. Nosso estudo indica que o SII pode ser utilizado para classificação de risco, em conjunto com a PCR-us, na predição da recorrência da pericardite ( Figura Central ).
