# Supplementary material for: Valor Preditivo do Índice de Inflamação Imune Sistêmica para a Recorrência de Pericardite Aguda Idiopática
Source: Arq Bras Cardiol. 2026 Mar 18;123(2):e20250437. [Article in Portuguese] doi: 10.36660/abc.20250437 (PMC13128187; doi:10.36660/abc.20250437)
Supplement: Translation

# Translation: Predictive Value of the Systemic Immune-inflammation Index for Recurrence of Idiopathic Acute Pericarditis

**DOI:** 10.36660/abc.20250437i

**Authors:** Tuba Unkun, Busra Guvendi Sengor

**Affiliations:** 1 Türkiye Department of cardiology, Kartal Kosuyolu Education and Research Hospital, Istanbul – Türkiye

**Keywords:** Systemic Immune-inflammation Index, Pericarditis, C-reactive Protein

## Abstract

**Background:**

Identifying patients at increased risk of recurrence of acute pericarditis remains challenging. High-sensitivity C-reactive protein (hs-CRP) can be used to predict recurrence, but it has certain limitations.

**Objectives:**

This study aimed to evaluate the association between a novel biomarker, the Systemic Immune-Inflammation Index (SII), and the recurrence of acute pericarditis.

**Methods:**

This retrospective cohort study included 110 patients diagnosed with acute idiopathic pericarditis, the primary endpoint of the study was to investigate Recurrent Pericarditis (RP) during the 18 month follow-up period. Patients were divided into two groups based on whether or not they developed recurrent pericarditis. Physical examination, electrocardiography, echocardiography, and laboratory test results of these patients were evaluated. P < 0.05 was considered statistically significant.

**Results:**

The mean age of the patients was 42.8 ± 15.2 years, and 42.7% were male. The group with RP had significantly higher leukocyte counts (8.7 ± 3.2 vs. 12 ± 4.7; p = 0.001), neutrophil counts (5.4 ± 2.7 vs. 9 ± 4.5; p < 0.001), hs-CRP levels (29.1 ± 40.9 vs. 49.5 ± 62.2; p = 0.048), and neutrophil-to-lymphocyte ratio (2.9 ± 2.5 vs. 6 ± 2.8; p < 0.001). SII (783.2 ± 770.2 vs. 1664.2 ± 1086.6; p < 0.001) was also significantly higher in patients with recurrence. Both SII and hs-CRP were independently associated with recurrence (p < 0.001 and p < 0.05, respectively).

**Conclusion:**

SII and hs-CRP were found to be independent predictors for recurrence in acute pericarditis patients during the 18 month follow-up. Similar to hs-CRP, SII may also be used as an important parameter in predicting recurrent pericarditis.

## Introductıon

Acute pericarditis is the most common pericardial disease seen in clinical practice.^1 , 2^At least two of the following four criteria are required for a diagnosis: chest pain, pericardial friction rub, abnormal electrocardiographic findings (PR depression and widespread ST elevation), and new or worsening pericardial effusion. Additional supportive findings include elevated inflammatory markers, such as High Sensitive C-Reactive Protein (hs-CRP) and Erythrocyte Sedimentation Rate (ESR), as well as inflammation detection using imaging techniques such as Cardiac Magnetic Resonance (CMR) or Computed Tomography (CT).^3^Complications of pericarditis include cardiac tamponade, constrictive pericarditis, and most commonly, recurrent pericarditis.^4 , 5^Acute pericarditis is an inflammatory condition that affects the pericardium. established. Elevated hs-CRP levels have been associated with pericarditis recurrence and adverse cardiac outcomes.^6 , 7^The search for reliable biomarkers capable of predicting recurrence in clinical practice is still ongoing.

Recently, the Systemic Immune-inflammation Index (SII), a simple yet comprehensive indicator, has gained attention.^8^SII reflects the overall inflammatory and immunological state by incorporating three biomarkers: platelets, neutrophils, and lymphocytes.^9^The SII index is commonly used for predicting the presence and prognosis of many malignancies.^10^Recent studies have also showed that SII is linked to clinical prognosis in a variety of cardiovascular illnesses.^11^However, the relationship between SII and the prognosis of acute pericarditis patients has not yet been demonstrated. The purpose of this study is to investigate the relationship between recurrent pericarditis following acute pericarditis and the SII index.

## Method

### Study population and design

This retrospective cohort study was conducted at Kartal Koşuyolu High Specialty Training and Research Hospital, Istambul, Turkey, from January 2021 to March 2024. The study involved 331 patients diagnosed with acute pericarditis. 150 patients were excluded from the study due to insufficient follow-up time after diagnosis. 15 patients with an ejection fraction (EF) less than 53-54 % (< 53% for men and < 54% for women)^3^or positive troponin levels, and 56 patients with specific underlying causes (such as malignancies, tuberculosis, autoimmune diseases, purulent pericarditis, post-myocardial damage or after myocardial infarction, renal failure (Glomerular Filtration Rate, GFR <60 mL/min), hepatic disorders – two-fold higher serum Aspartate Aminotransferase (AST) and >80 U/L or Alanine Aminotransferase (ALT) >112 U/L activity than normal limits. After exclusions study group consist of 110 patients diagnosed with acute pericarditis ( Figure 1 ). The study was authorized by the local ethics committee.

**Figure 1:** – Flow diagram of the studied population with acute pericarditis.

All clinical and demographic data, including age, sex, and medical history, were obtained from the hospital’s digital health record system. All patients experienced their first episode of pericarditis, and the diagnosis was established based on the presence of at least two of the four criteria outlined in the latest guidelines: pericardial chest pain, electrocardiographic abnormalities (new widespread ST elevation or PR depression), pericardial effusion, and pericardial friction rub.^3^Admission electrocardiograms, chest X-rays, echocardiograms, and blood test results were retrieved from the hospital records. Blood tests included complete blood count, hs-CRP, high-sensitivity troponin I, serum biochemistry, thyroid function tests, and screening for specific diseases (such as autoimmune disorders and tuberculosis). The Neutrophil-to-Lymphocyte Ratio (NLR) was calculated as the ratio of neutrophil count to lymphocyte count in peripheral blood samples. The SII was calculated as SII = Platelet count (PLT) × NLR.^9^Complete blood counts were determined using an autoanalyzer (Horiba ABX Diagnostics, France), and C-reactive protein levels were measured using the nephelometric method (Beckman Coulter).

Echocardiographic images were retrieved from the hospital’s digital health record system. Cardiac tamponade was diagnosed based on a combination of clinical symptoms (such as pulsus paradoxus, hypotension, and tachycardia) together with echocardiographic evidence. Pericardiocentesis was performed in patients with severe pericardial effusion accompanied by hemodynamic instability or when clinical findings suggested bacterial or neoplastic pericarditis.

According to guideline recommendations, Nonsteroidal Anti-inflammatory Drugs (NSAIDs) or aspirin were used as the first-line treatment at the maximum tolerated dose.^3 , 12^The maximum dose was administered for 1–2 weeks, and if hs-CRP levels normalized, the dose was tapered over 3–4 weeks. In all cases, colchicine 0.5 mg twice daily was prescribed for 3 months, with adjustments based on creatinine clearance, body weight, and age.^3 , 1 3^No patients received corticosteroid therapy. Recurrent pericarditis was defined as the presence of recurrent chest pain in addition to at least one of the following: fever, pericardial friction rub, ECG abnormalities, evidence of pericardial effusion, increased leukocyte count, or elevated hs-CRP.^3^

All patients included in the study were followed approximately 18 months from the time of diagnosis. Approximately 10 days after discharge, they were followed monthly for the next 3 months, and thereafter every 3 months up to 18 months. Clinical visits included physical examinations, blood test were including complete blood count and CRP, ECG, and echocardiographic evaluations. The study endpoint was the occurrence of the first recurrence of pericarditis. Patients were divided into two groups based on whether or not they developed recurrent pericarditis.

### Statistical analysis

The study was approved by the Local Ethics and Clinical Investigation Committee and had conformed to the principles embodied in the Declaration of Helsinki. Statistical analyses were performed by using Statistical Package for the Social Sciences (SPSS; Version 26.0, IBM). Continuous variables were presented as mean and standard deviation due to the normality of the data. Categorical variables were presented by absolute and relative frequencies. Assumption of normality of data were evaluated by Kolmogorov–Smirnov test. The comparison between groups was made using the Student’s t-test for independent samples or chi-Square, as appropriate. Enter model was used in multiple logistic regression. Results of these models were interpreted in terms of adjusted odds ratios (aORs) with associated 95% confidence intervals (95% CIs). Receiver operating characteristic curve (ROC curve) analysis was used to find the optimal cut-off for hs-CRP and SII in recurrence of pericarditis Results were evaluated with a confidence interval of 95%, and p < 0.05 was considered statistically significant.

## Results

Patients were devided in two groups according to recurrence the study population consisted of 110 patients (47 males, 42.7%) and mean age of the patients was 42.8±15.2years. The characteristics of the patients are presented in Table 1 .

**Table 1:** – Demographic and clinical characteristics of the patients

Variables		No recurrence (n=81)	Recurrence (n=29)	p values
Age (years)		42.19 ±15.5	42 ±15.2	0.956^1^
Gender (n)	Man	34	13	0.790^2^
	Woman	47	16	
ECG (n)	No change	21	9	0.596^2^
	Change	60	20	
Pericardial effusion (n)	Mild	66 (81.4%)	15 (51.7%)	0.006*
	Moderate	4 (4.9%)	5 (17.2%)	
	Severe	11 (13.7%)	9 (31.1%)	
Presence of chest pain (n)		70	27	0.105^2^
Pericardial friction rub (n)		17	2	0.065^2^

^1^Independent samples T test,^2^Chi square test, *p<0.05; ECG: electrocardiogram.

All patients received the same treatment (NSAIDs and colchicine). There was no difference in age, gender, chest pain, and ECG changes between the groups. The leukocytes and neutrophyl counts were higher in the recurrence group (p<0.001 Table 2 ). SII, NLR and hs-CRP were also found to be significantly higher in patients with recurrence (p:<0.001, p:<0.001, p:0.048). Other findings are presented in Table 2 .

**Table 2:** – Comparisons of laboratuary variables between the patients with and without recurrence

Variables	No recurrence (n=81)	Recurrence (n=29)	p values
Hs-CRP (mg/L)	29.1 ±40.9	49.5 ±62.2	0.048
Leukocyte (cells/µL)	8.7 ±3.2	12 ±4.7	0.001*
Hemoglobin (g/dl)	13.2 ±1.7	12.8±2	0.306
Platelet (cells/µL)	267.4 ±86.8	312.2 ±126.3	0.086
Neutrophil (cells/µL)	5.4± 2.7	9 ± 4.5	<0.001**
Lymphocyte (cells/µL)	2.3±1.13	2.5±3.2	0.786
SII	783.2 ± 770.2	1664.2 ±1086.6	<0.001**
Neutrophil Lymphocyte ratio	2.9 ± 2.5	6 ± 2.8	<0.001**

Independent samples T test, *p<0.05, **p<0.001; SII: Systemic Immune-Inflammation Index.

In regression analysis the co-founding variables included SII, pericardial effusion, hs-CRP. SII was independently associated with recurrence. (Odds Ratio (OR) = 1.4 with %95 CI [0.96-1.54] p:0.002). Other variables are presented in Table 3 .

**Table 3:** – Logistic regression analysis to predict recurrence

Variables	Odds Ratio	95% CI	p values
SII	1.4	0.96-1.54	0.002
Pericardial effusion	1.2	0.78-2.7	0.23
Hs-CRP	0.9	0.98-1.009	0.8

*SII: Systemic Immune-Inflammation Index.*

In ROC analysis ( Figure 2 ), SII was found the most associated parameter with recurrence. The cut-off point determined for SII in the diagnosis of recurrence is 462.5 with 82.8% sensivity and 40% specifity . The ROC analysis results are shown in Figure 2 . (AUC:0.751, CI:0.630-0.872, p:0.001).

**Figure 2:** – Receiver operating characteristic (ROC) analysis.

## Discussion

Our study demonstrated that SII is simply calculated and cheap parameter for predicting recurrence in acute pericarditis patients. Acute pericarditis is the most frequent presentation of pericardial disorders and causes significant morbidity. Recurrent pericarditis, the most common complication following acute pericarditis, significantly impacts the patient’s quality of life.^3 , 14^Identifying patients prone to recurrence is challenging.

In clinical practice, identifying patients with a high risk of recurrence among those diagnosed with a first episode of acute pericarditis is crucial, as recurrent pericarditis leads to repeated attacks and chronic disease progression.^15^However there is not specific marker or diagnostic process for detecting this patients . Patients with a high risk of recurrence may benefit from close monitoring and intense medical treatment. Also they could additionally benefit from expert imaging modalities (such as CMR) prior to discontinuing medication. CMR, with its ability to show ongoing inflammation in the tissue, will be beneficial for determining the treatment duration.^3^

Patients at high risk of recurrence after a first episode of acute pericarditis have not been adequately investigated to date. According to previous studies, colchicine use during the initial attack has been shown to be the most effective factor in reducing recurrence. Conversely, the use of corticosteroids, specific etiologies of acute pericarditis, inadequate treatment, and the presence of pericardial effusion have all been associated with higher recurrence rates.^3 , 5 , 16 - 20^

Inflammatory markers are used to predict the recurrence in acute pericarditis. Hs-CRP can be used to diagnose as well as assess therapy response.^6 , 21^Imazio et al.^7^found that sustained hs-CRP rise could predict pericarditis recurrence. Studies have indicated that NLR, an inflammatory marker, is more effective than hs-CRP in predicting pericarditis recurrence.^22^A recent study has shown that D-dimer is a potential biomarker for prognostic assessment in acute and complicated pericarditis.^23^In another study, the Infla Score – developed by combining various inflammatory markers – was found to be effective in predicting pericarditis recurrence.^24^SII has been found in studies to be more potent inflammatory marker than NLR or PLT alone, with a better prognostic value.^25^In this study, we found that SII was more significantly linked with the likelihood of recurrent pericarditis than NLR, PLT, and hs-CRP alone, suggesting that SII may serve as a more reliable predictor of the patients’ inflammatory status, resulting in higher predictive reliability for recurrent pericarditis.

We hypothesized that the use of the SII – a simple, more comprehensive, and less variable index that simultaneously incorporates multiple inflammatory biomarkers – may serve as a supportive parameter to CRP, which is routinely used in follow-up, and may also provide significant prognostic value in detecting underlying active inflammation in acute and recurrent pericarditis. Comprising neutrophils, lymphocytes, and platelets, SII has recently been developed as a comprehensive biomarker of systemic inflammation.^9^SII was originally found to be a powerful prognostic factor for patients with hepatocellular carcinoma.^8^SII is now widely employed as a predictive and prognostic marker in a variety of disorders, including cancer, cardiovascular disease, and kidney disease.^26 - 28^

SII has gained popularity in recent years due to its role as an inflammatory marker, similar to hs-CRP. It is a simple parameter suitable for routine use, and its variability is lower than that of hs-CRP. In our study, SII was identified as an independent predictor of recurrent pericarditis, suggesting that it may serve as a valuable parameter in clinical practice. When used in conjunction with hs-CRP, rather than as a substitute, SII could improve the prediction of patients at risk for recurrent pericarditis.

To the best of our knowledge, no research has been conducted in acute pericarditis individuals to explore the association between SII and prognosis. In our study, SII exceeded hs-CRP in predicting pericarditis recurrence.

This study has several limitations. First, the relatively small sample size of 110 patients may limit the generalizability of the findings to broader clinical settings and more diverse patient populations. In addition, the retrospective design of the study meant that certain variables – such as treatment adherence, symptom duration, and consistency of follow-up – could not be fully standardized or controlled. The absence of serial measurements of laboratory parameters also represents a significant limitation.

Furthermore, only patients with isolated pericarditis were included. Myopericarditis and perimyocarditis, which involve concurrent pericardial and myocardial inflammation, may present with similar clinical symptoms and initial findings, increasing the risk of misclassification in the absence of advanced imaging. However, because advanced imaging techniques (e.g., cardiac MRI) are not routinely available in all clinical settings, these patients were excluded. While this approach allowed for the selection of a lower-risk and more homogeneous study population, it limits the generalizability of the findings to the broader spectrum of pericardial disease. In addition, out of a total of 331 patients, only 110 who met the predefined inclusion criteria and had sufficient clinical data and follow-up information were included in the analysis. Since detailed clinical, laboratory, or treatment data were not systematically collected for the 221 excluded patients, a separate analysis of this group could not be performed. This may have introduced a potential selection bias and reduced the external validity of the study.

Finally, although all patients were prescribed NSAIDs and colchicine at discharge in accordance with European Society of Cardiology guidelines, post-discharge adherence to treatment could not be systematically monitored due to the retrospective nature of the study. Methods commonly employed in randomized controlled trials, such as telephone follow-ups, face-to-face interviews, or pill counts, were not applied. Consequently, treatment uniformity and the assessment of therapeutic efficacy represent additional limitations.

SII was found to be an independent predictor for pericarditis recurrence in patients with acute pericarditis during the follow-up. Our study indicates that SII could be used for risk classification in addition to hs-CRP for prediction of recurrence pericarditis ( Central Illustration ).
